# Misdiagnosis of adult primary hemophagocytic lymphohistiocytosis as NK/T‐cell lymphoma: A case report

**DOI:** 10.1002/jha2.564

**Published:** 2022-10-06

**Authors:** Qi Kong, Jingshi Wang, Yanlin Zhang, Junxia Hu, Mingzhu Yu, Lin Wu, Zhao Wang

**Affiliations:** ^1^ Department of Hematology Beijing Friendship Hospital Capital Medical University Beijing China; ^2^ Department of Pathology Beijing Friendship Hospital Capital Medical University Beijing China

**Keywords:** central nervous system disease, genome sequencing, lymphoma, perforin, primary hemophagocytic lymphohistiocytosis

## Abstract

We reported a case of a 19‐year‐old male patient with central nervous system symptoms as the main clinical manifestations, and multiple intracranial and abdominal occupying lesions visualized by imaging examinations, who was initially misdiagnosed as NK/T‐cell lymphoma but poorly responsive to the treatment. Finally, he was diagnosed as familial hemophagocytic lymphohistiocytosis type‐2 by genome sequencing, perforin test and pedigree study. The patient survived well after allogeneic hematopoietic stem cell transplantation. Central nervous system symptoms could be the main clinical manifestations in patients with primary hemophagocytic lymphohistiocytosis , whose early‐stage manifestations of blood system were usually atypical, easily leading to misdiagnosis. In clinical practice, primary hemophagocytic lymphohistiocytosis should be considered in patients with central nervous system symptoms and unknown causes. The combination of rapid immunological function test and genome sequencing contributes to the diagnosis of primary hemophagocytic lymphohistiocytosis.

## 1 INTRODUCTION

IPrimary hemophagocytic lymphohistiocytosis (pHLH) is a severe inflammatory response disorder induced by abnormal regulation of immune system, which is regulated by genetic defects. It is a rapidly progressive and highly lethal disease. Therefore, timely diagnosis and treatment are crucial for pHLH patients. Adult cases of pHLH are rarely reported, and the vast majority (>90%) develops pHLH before 2 years of age [1]. However, the onset age is not a decisive factor for the diagnosis of pHLH. Family history of pHLH is frequently detected in affected people, and they usually carry mutations of the *PRF1*, *UNC13D*, *STX11*, *STXBP2*, *SH2D1A*, *BIRC4*, *Rab27a*, *LYST*, *ITK*, *CD27*, and *MAGT1* gene [2]. In this case report, we reported an adult pHLH patient who was initially misdiagnosed as NK/T‐cell lymphoma and reviewed the diagnosis of pHLH and clinical features of central nervous system‐hemophagocytic lymphohistiocytosis (CNS‐HLH).

## 2 CASE PRESENTATION

**FIGURE 1:**
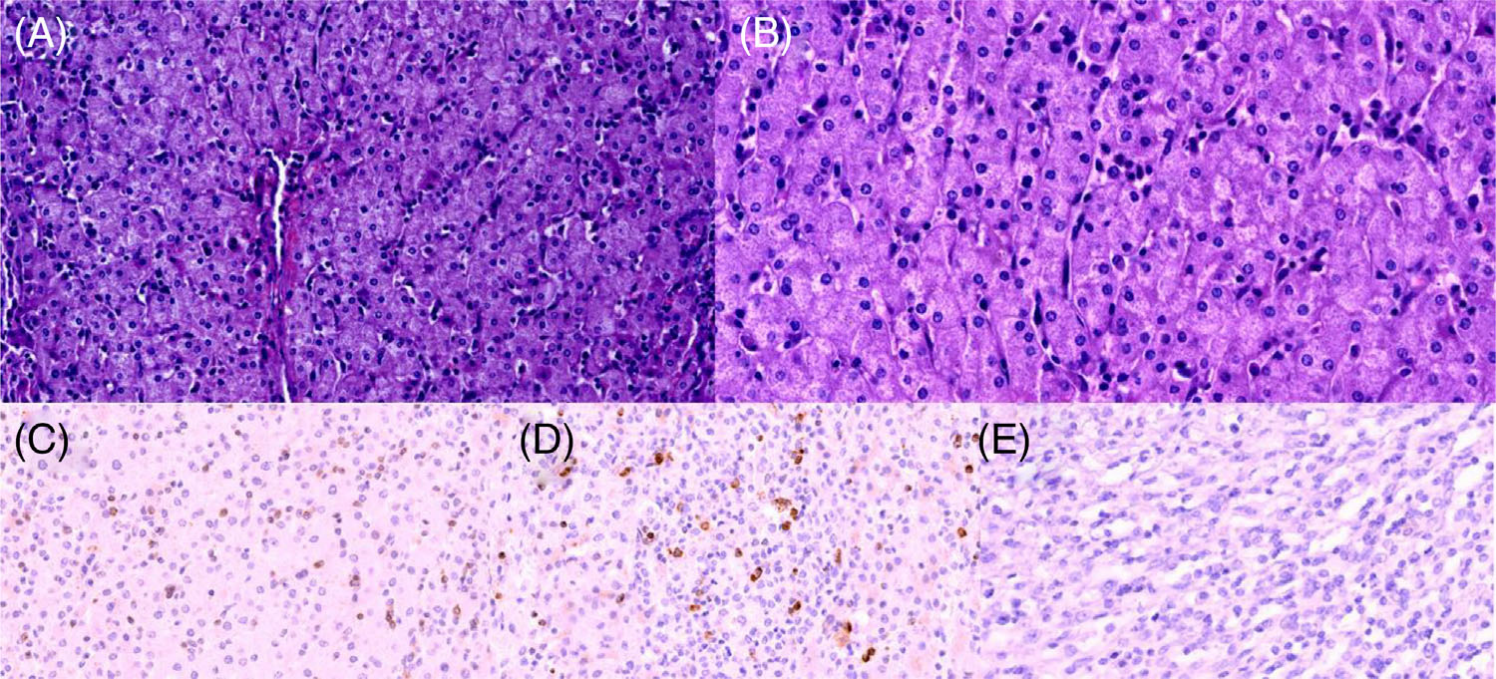
Pathological sections of liver. (A) The liver tissue structure is not damaged, and lymphocytes are scattered in the hepatic sinuses. (B) The volume of lymphocytes in hepatic sinuses is small, and the atypia is not obvious. (C) CD3 showed that lymphocytes were scattered positive. (D) GrB showed that lymphocytes were scattered positive. (E) EBER in situ hybridization showed negative. EBER, Epstein‐Barr encoding region

**FIGURE 2:**
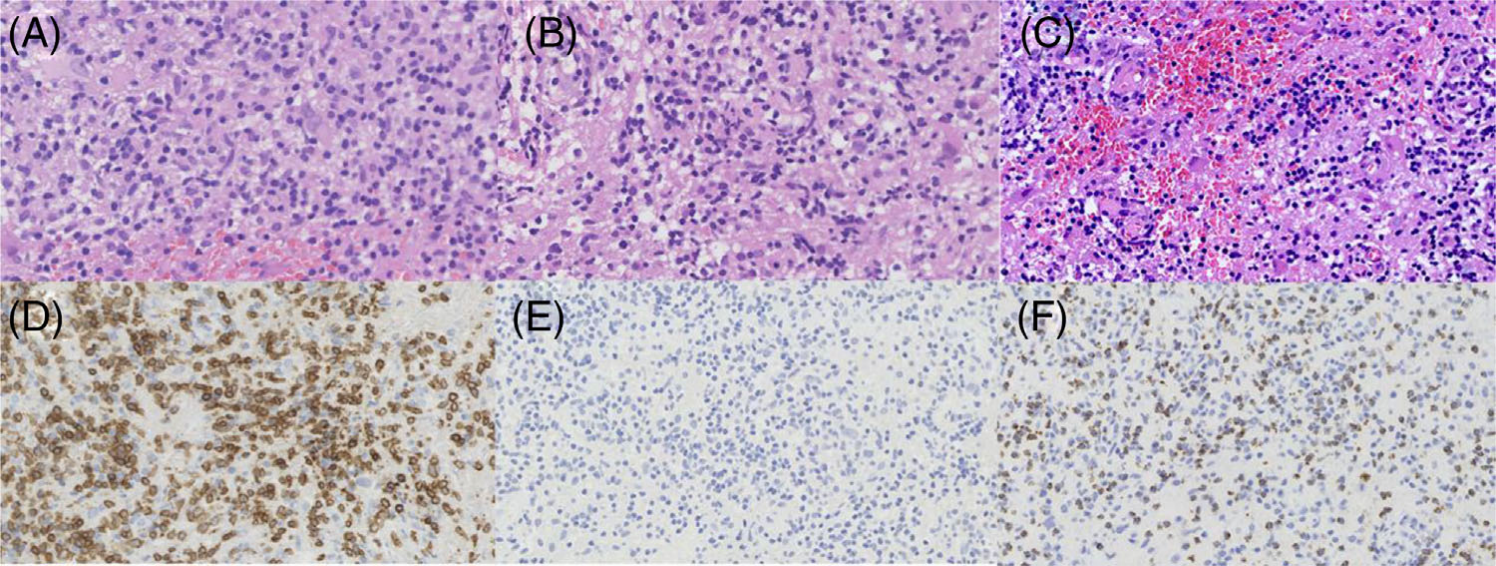
Pathological sections of left cerebellar hemisphere. (A) No structural damage is found in brain tissue, and focal hemorrhage can be seen. Scattered lymphocyte infiltration can be seen in the stroma. Lymphocytes are small and medium in size, slightly irregular in morphology, and nucleoli and mitosis are not easy to see. (B) It shows scattered and small clusters of lymphocytes, which surround the blood vessels. (C) This image shows multifocal hemorrhage. (D) CD3 showed that lymphocytes were strongly positive. (E) EBER in situ hybridization showed negative. (F) TIA1 showed that lymphocytes were strongly positive. EBER, Epstein‐Barr encoding region

A 19‐year‐old male patient developed recurrent fever on August 11, 2020, with a maximum body temperature of 38.8°C. Peripheral blood cells and pulmonary compuerized tomography (CT) image were normal. The patient was poorly responsive to the antibiotic treatment. Then, he presented facial paresthesias and mild cognitive and thinking abnormalities on September 20, 2020. Liver function test showed: alanine aminotransferase (ALT), 221 U/L; aspartate transaminase (AST), 333 U/L; total bilirubin (TBil), 109.3 µmol/l; direct bilirubin (DBil), 88.8 µmol/l; lactate dehydrogenase (LDH), 507 U/L. Serum ferritin (SF), 28642 ng/ml. Positron emission tomography‐computed tomography (PET‐CT) examination showed: (1) Multiple occupying lesions with increased fluorodeoxyglucose (FDG) metabolism in the intracranial and abdominal cavity, and liver. (2) Splenomegaly. (3) Multiple enlarged lymph nodes in the bilateral neck, mediastinum, axilla, and the internal mammary area with increased FDG metabolism, considering lymphoma. Pathology of liver biopsy (Figure 1) showed small lymphoid cells with slightly irregular karyotypes infiltrating in some areas. Flow cytometry showed cell populations expressing abnormal T and NK antigens. Pathology of the excisional lesions in left cerebellar hemisphere (Figure 2) showed lymphocytes infiltrated and vascular sleeve formed in some areas; flow cytometry showed a few abnormal lymphocytes expressing abnormal T and NK antigens. The patient was considered as NK/T cell lymphomal. The patient was successively medicated with pagaspargase, toripalimab[3, 4], methotrexate, vincristine, and etoposide and chidamide[5, 6]. During the hospitalization from October 7, 2020 to November 16, 2020, the patients were treated with glucocorticoid every day and given antibacterial and antifungal infection treatment. However, the patient still presented recurrent fever, further increased ferritin and deteriorated liver function, enlarged spleen, and newly emerging pancytopenia.

**FIGURE 3:**
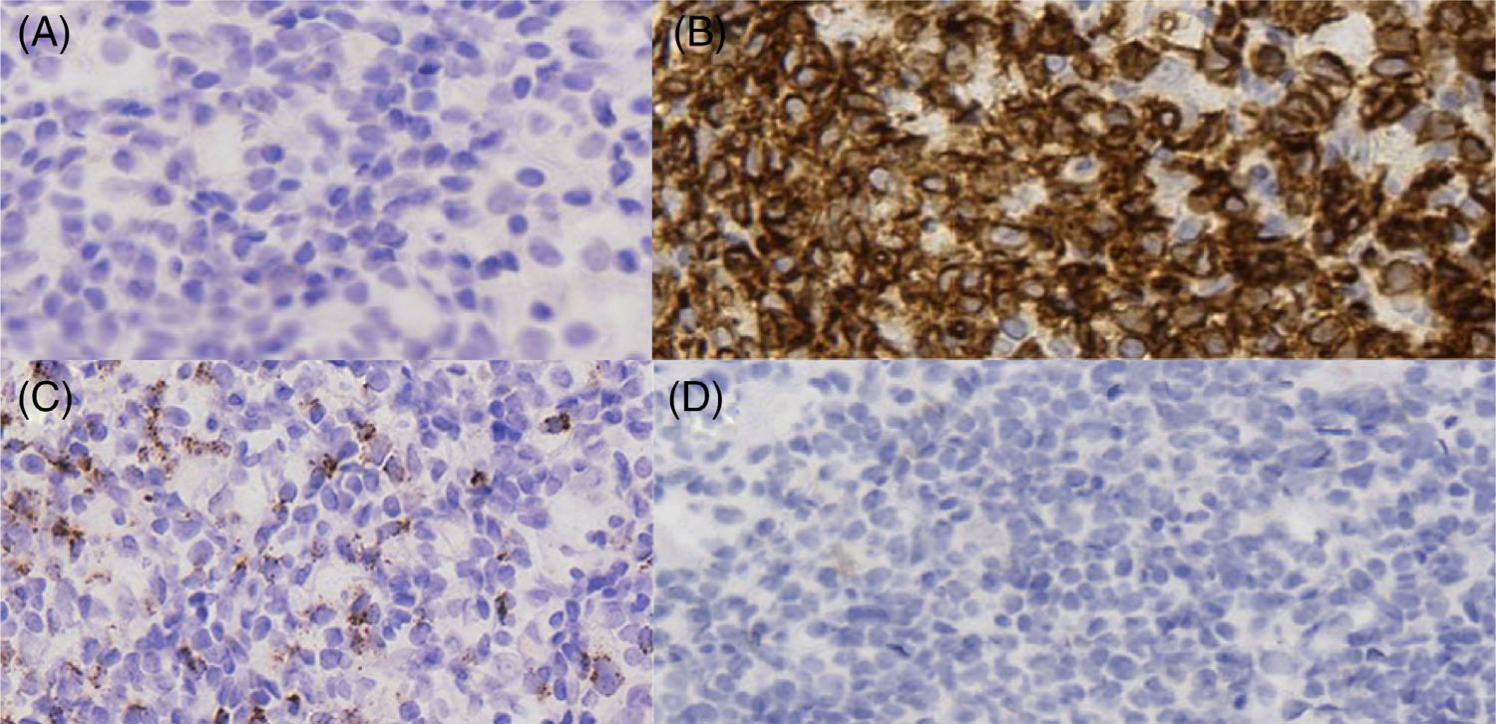
Pathological sections of the right axillary lymph node. (A) Lymphocytes are small and medium in size, slightly irregular in morphology, and nucleoli and mitosis are not easy to see. (B) CD3 showed that lymphocytes were strongly positive. (C) GrB showed that lymphocytes were scattered positive. (D) EBER in situ hybridization showed negative. EBER, Epstein‐Barr encoding region

**FIGURE 4:**
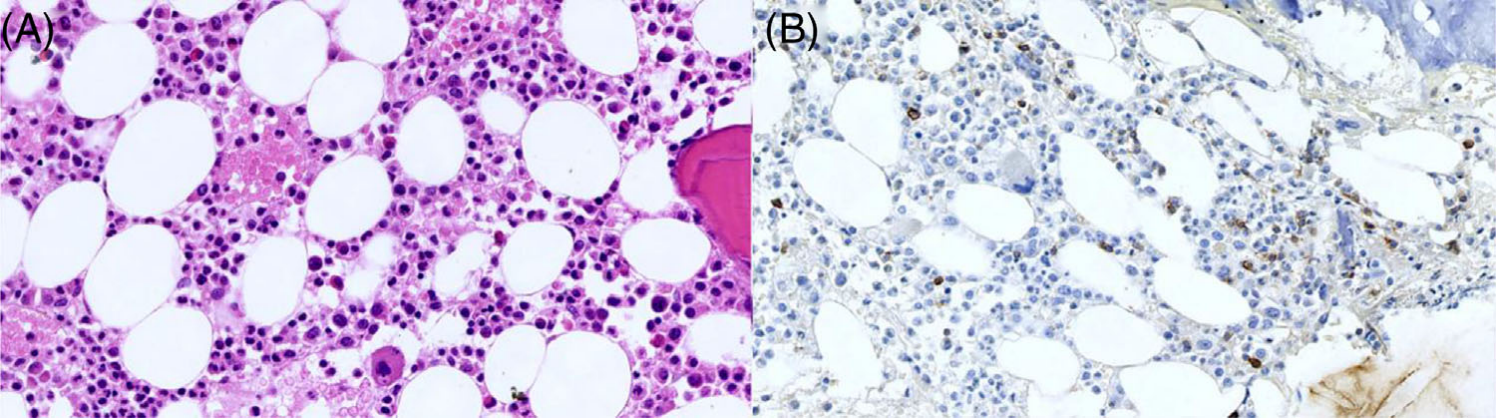
Pathological sections of bone marrow. (A) There is normality in the hematopoietic structure of bone marrow, showing a hyperplastic bone marrow image, with scattered small lymphocyte infiltration. (B) CD3 showed that lymphocytes were scattered positive

**FIGURE 5:**
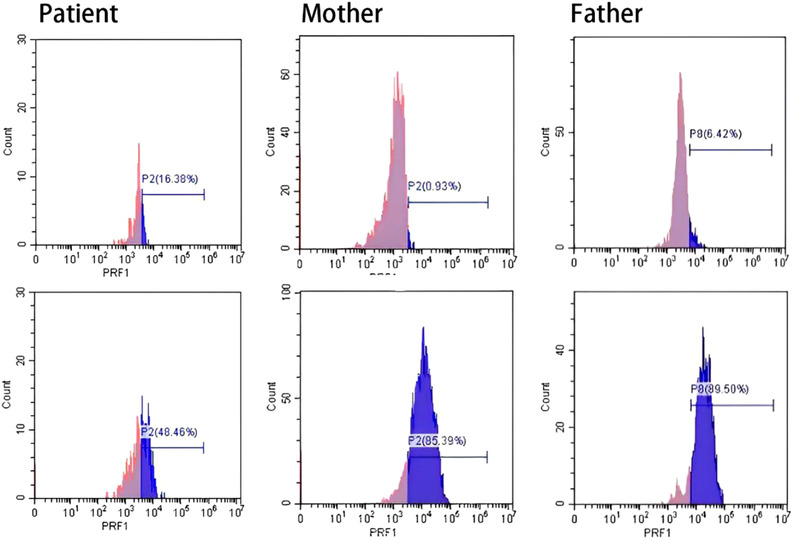
Flow cytometry plots of perforin in NK cells

The patient was admitted to our hospital on November 20, 2020. Peripheral blood cells test showed: white blood cell count (WBC), 1.19 × 10^9^/L; hemoglobin, 85 g/L; platelet count, 92 × 10^9^/L. Blood biochemistry test showed: ALT, 239 U/L; AST, 339 U/L; alkaline phosphatase, 2964 U/L; Gamma‐glutamyl transferase, 1136 U/L; TBil, 142.5 µmol/L; DBil, 67.09 µmol/L; triglyceride, 21.05 mmol/L; LDH, 860 U/L. Coagulation function test showed: fibrinogen 0.59 g/L. SF, 39220 ng/ml. sCD25, 27622 pg/ml (reference range < 6400 pg/ml). NK cell activity was 12.73% (reference range ≥ 15.11%). Bone marrow morphology showed hemophagocytic phenomenon. Antinuclear antibodies, autoimmune hepatitis antibodies, and immunoglobulins were normal. EBV‐DNA and CMV‐DNA levels were both below the detective limit. The detectable antigens and/or antibodies against Schistosoma japonicum, Clonorchis sinensis, hepatic hydatid, Doleishmania, Brucella, dengue virus, and hantavirus were all negative. Test of EBV‐DNA, Cryptococcus, filarial antigen, cysticercosis antibody, Sparganum mansoni antibody and Trichinella antibody in cerebrospinal fluid (CSF) were all negtive, and bacterial and fungal culture of pathogen in CSF were also negative. The “next‐generation” sequencing of pathogen DNA of CSF was also negtive. The CSF pressure was normal, with upregulated CSF proteins, and no abnormal cell populations were detected in cell smear and immunophenotyping. Biopsy pathology of the right axillary lymph node (Figure 3) showed slightly irregular T cells with undetermined nature. Bone marrow biopsy pathology (Figure 4) showed reactive lymphoid hyperplasia. Whole‐exome sequencing showed genetic defects of familial hemophagocytic lymphohistiocytosis (FHL) with two heterozygous mutations of p.V61A missense mutation (c.182T>C) and p.T450M missense mutation (c.1349C>T) in the coding sequence of the *PRF1* gene. The expression rate of perforin in NK cells was 32.08% (reference value ≥ 81%) (Figure 5), and the function of cytotoxic cell degranulation (CD107a) and the expression rate of granzyme B, SAP protein, XIAP protein, and munc13‐4 protein were normal. Through pedigree study, the patient was the only child in his family, and both parents were healthy. His mother did not have a history of abnormal pregnancy. No family history of hematological diseases and immunodeficiency was reported. Genome sequencing showed a heterozygous missense mutation of p.V61A in the *PRF1* gene in the patient's father and a heterozygous missense mutation of p.T450M in the *PRF1* gene in the patient's mother. The perforin test in both parents was normal (Figure 5). According to the HLH‐2004: Diagnostic and therapeutic guidelines [7], the patient was diagnosed as FHL type‐2 (FHL‐2) and intervened with four courses of dexamethasone, etoposide, and doxorubicin liposome (DEP) chemotherapy [8]. Partial response was achieved in this case after chemotherapy.

The patient underwent haploidentical hematopoietic transplantation from the mother donor in January 2021. At 21 days postoperatively, the bone marrow chimerism rate achieved 99%, with a complete donor chimerism. At 43 days postoperatively, SF, sCD25, NK cell activity, and expression of perforin were all returned normal. Genome sequencing revealed only a heterozygous missense mutation of p.T450M in the *PRF1* gene from the patient's mother.

**FIGURE 6:**
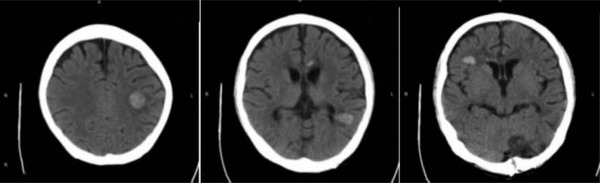
CT images of the patient's brain before transplantation. Multiple high‐density nodules and masses with peripheral edema in bilateral semiovale center, corpus callosum, bilateral paraventricular areas, right frontal lobe, and left cerebellar hemisphere

**FIGURE 7:**
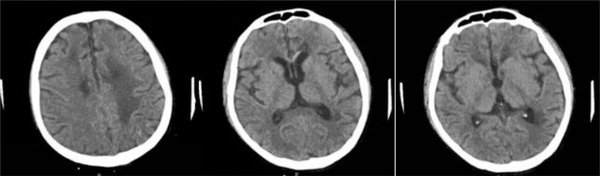
CT images of the patient's brain 3 months after transplantation. The nodules in the brain parenchyma became significantly smaller than before

Six months after the transplantation, the patient was re‐examined without obvious advanced neurological dysfunction, and the CSF pressure, protein levels, WBC, and immunophenotyping were all normal. PET‐CT images showed significantly reduced intracranial and abdominal occupying lesions. No enlargement of the liver, spleen, and lymph nodes were observed, nor as increased metabolism. The comparison of brain images before and after transplantation is shown in Figures 6 and 7.

## 3 DISCUSSION

Hemophagocytic lymphohistiocytosis is a severe inflammatory response syndrome. Macrophages and T lymphocytes are activated and proliferated excessively, caused by primary or secondary factors,resulting in cytokine storm. Typical histopathological manifestations of HLH include extensive accumulation of lymphocytes and mature macrophages, and sometimes accompanied by hemophagocytic phenomena, especially in the spleen, lymph nodes, bone marrow, liver, and CSF. HLH can be divided into two categories: primary (pHLH) and secondary HLH (sHLH). pHLH includes FHL, immunodeficiency syndrome‐related HLH, and Epstein‐Barr (EB) virus‐driven HLH, of which FHL is the most‐common subtype. sHLH is closely related to infection, tumors, rheumatic diseases, etc. Malignancy‐related HLH is mainly observed in patients with hematological tumors, among which lymphoma‐related HLH is the most‐common one, especially in cases of NK/T‐cell lymphoma [9]. Considering the clinical manifestations of multiple intracranial and abdominal masses, enlargement of the liver, spleen and lymph nodes, suspected lymphoma by PET‐CT, and cell populations expressing abnormal T and NK antigens by immunophenotyping of the liver and brain, the patient was initially misdiagnosed as NK/T‐cell lymphoma in the other hospital. However, the patient was poorly responsive to the antilymphoma therapy. Pathological diagnosis is always the gold standard for lymphoma. During the course of the disease, a clear pathological diagnosis of the liver, brain, lymph nodes, and bone marrow in this patient was thoroughly scant. Therefore, we considered that the etiology of HLH may not be lymphoma, and the abnormal T and NK cells may be reactive hyperplasia caused by HLH.

Primary HLH used to be diagnosed according to the young age and positive family history, and as a result, adult patients are often diagnosed with sHLH. Since the end of the last century, FHL‐related defective genes have been discovered successively, and the HLH‐2004: Diagnostic and therapeutic guidelines further recommended that gene sequencing is the gold standard for diagnosing pHLH [7]. Later, more adult pHLH patients have been reported. The patient has p.T450M missense mutation in the coding sequence of the *PRF1* gene, which has been reported in FHL‐2 before[10, 11]. He was transferred to our hospital and finally diagnosed as pHLH by whole‐exome sequencing (WES), indicating the clinical significance of WES in adult patients, especially those with unclear etiology, recurrent or progressive disease.

The adult onset of this patient may be attributed to the genetic susceptibility due to the mutations, although it was not enough to cause clinical manifestations. A previous study has shown that IFNγ^−/−^ mice can survive, and clinical symptoms of HLH are only present in those infected with lymphocyte choriomeningitis virus [12]. It is suggested that in addition to inherent immune deficiencies, environmental factors like viral infection are also involved in the pathogenesis of pHLH. From the perspective of genetics, gene mutations in some atypical regions barely affect the function of encoded proteins. Although they can cause cytotoxic cell dysfunction, clinical manifestations can be absent. There may be other inducements leading to the onset of pHLH. We thoroughly asked the personal history of this patient and learned that he had traveled to Daocheng and Ganzi in Sichuan 3 months before the onset, and drank the grassy stream water. However, we conducted a series of measurable pathogen examinations in the patient ended up with no positive results. In addition, disease onset is linked with the impairment degree of cytotoxic function caused by mutations, and the missense mutation in the FHL‐deficient gene is associated with late onset in some cases [13]. This patient had a heterozygous missense mutation, which was consistent with previous findings. FHL may be induced by different reasons and present varied clinical manifestations. However, gene defect is its essential cause. According to the current diagnostic criteria, the early clinical manifestations of HLH may be atypical (e.g., CNS involvement) that cannot contribute to the diagnosis. Ammann et al. conducted a large‐scale cohort study, and they found that 17% of FHL patients have atypical clinical manifestations or do not fully meet the five of eight diagnostic criteria [14]. Therefore, those with CNS manifestations and unknown etiology should be suspected of pHLH, and detection of pHLH gene defects is recommended.

Gene mutation affects the function of cytotoxic cells by changing the functional proteins it encodes. In clinical applications, genome sequencing is time‐consuming, while cytotoxicity assay is convenient and fast, which is of great significance to the diagnosis and classification of pHLH, as well as the selection of optimal donors. The *PRF1* gene is closely linked with FHL‐2, and its encoded protein perforin exists in NK cells and cytotoxic T lymphocytes. Mutations of the *PRF1* gene result in decreases of the expression, activity, and stability of perforin. As a result, pore formation in cell membranes of target cells is impaired, leading to reduced killing effect on target cells and abundant accumulation of inflammatory factors, and finally leading to HLH [15]. We detected the positive expression of perforin in this patient, which was reduced in NK cells. Although the detection of perforin has not been listed in the HLH‐2004: Diagnostic and therapeutic guidelines, it has been validated with a high accuracy in the differential diagnosis of pHLH. Rubin et al. [16] reported that abnormal expression of perforin is detected in 96.6% of HLH patients with *PRF1* gene mutations, and perforin test is more sensitive than that of cytotoxicity assay of NK cell with a comparable specificity. Therefore, the perforin test plays an important role in the indication and verifying of pHLH.

The incidence of CNS involvement in HLH cases ranges 18%–73% [17]. Kim et al. [18] reported that in their cohort involving 50 HLH patients, the proportion of CNS involvement is higher in FHL patients. Among pHLH population, FHL‐3 patients caused by mutations of the UNC13D gene present the highest proportion of CNS involvement up to 60%, which is only 36% in FHL‐2 patients caused by mutations of the PRF1 gene [19]. Currently, a consensus on the diagnosis criteria of CNS‐HLH is scant. It is generally considered that CNS‐HLH can be defined by abnormal findings in CSF and/or head MRI, with or without obvious neurological symptoms and signs [20]. However, clinical symptoms of CNS‐HLH are related to the location, scope, and degree of the intracranial lesions, which are often not specific. Horne et al. [20] reported that about one‐third of HLH patients have seizures, which can be considered as the most‐common neurological symptom. Notably, CNS symptoms can be the first symptoms in some HLH patients. Pastula et al. [21] reported a patient with progressive left hemiplegia and aphasia who is finally confirmed as HLH by autopsy. Thus, misdiagnosis easily occurs in CNS‐HLH patients with neurological symptoms as the first manifestation or even the single manifestation. Therefore, HLH should be highly suspected in those with CNS involvement, especially those with fulminant systemic diseases involving hemocytopenia and fever. Imaging and CSF abnormalities of CNS‐HLH also lack specificity. Low‐density foci in the white matter, ventricular dilatation, and local high‐density foci are the main abnormalities on CT scans. Head MRI is usually used to assist the diagnosis of HLH due to the unclear lesions on CT scans. The most‐common manifestations of MRI include multiple, bilateral lesions with demyelinating changes with low signal on the T1WI, and high signal on the T2WI and FLAIR. High signal on the diffusion weighted imaging can be detected in cases of cerebral hemorrhage or even necrosis [22]. CSF abnormalities in patients with CNS‐HLH mainly include mild to moderate increase in cell number (mostly lymphocytosis > 5 cells/µl) and/or increased protein content (>35 mg/dl). Besides, the increased CSF pressure detected during lumbar puncture may also be suggestive [17]. Notably, CSF abnormalities can also be detected in HLH patients with negative CNS symptoms [20]. In this case, the patient developed CNS symptoms during the course of the disease, manifesting as facial paresthesias and mild cognitive and thinking abnormalities, increased protein content in CSF, and multiple high‐density foci on brain CT scans, which were consistent with the definition of CNS‐HLH. Due to the nonspecificity of clinical symptoms, imaging, and CSF changes of CNS‐HLH, it is of signality to search for more specific and sensitive markers of CNS‐HLH.

Although the consensus in the diagnosis of CNS‐HLH is scant, it is generally agreed that the activated lymphocytes and macrophages infiltrating into the meninges and brain tissues are the main pathological manifestations of CNS involvement in HLH patients, which was initially proposed by Akima et al. in 1984 who analyzed neuropathology of six pHLH patients with CNS involvement [23]. They discovered that the severity of CNS‐HLH varies a lot, and lymphohistiocytic infiltration of the pia mater may be the mildest involvement, which can be gradually progressed to perivascular inflammation and even diffuse brain tissue infiltration with multifocal necrosis [23]. In 1996, Henter et al. [24] analyzed 23 children with CNS‐HLH, and they classified the degree of CNS involvement into three stages: Stage I: Infiltration of lymphocytes and histiocytes/macrophages into the meninges; stage II: Infiltration of brain parenchyma and the perivascular space in addition to the meninges; Stage III: Extensive brain parenchymal infiltration, especially white matter involvement, and signs of brain tissue necrosis, reactive astrogliosis, and demyelination [24]. The pathology of left cerebellar hemisphere mass in this patient showed lymphocytic infiltration in some areas, which was consistent with the pathological manifestations of CNS‐HLH.

## 4 CONCLUSION

Although great progress has been made on the diagnosis and treatment of pHLH, those with atypical manifestations are easily misdiagnosed due to the individualized differences. Positive findings on PET‐CT scans may mislead the diagnosis. Pathological examination, as the gold standard, usually cannot set the tune once and for all or even leads to misdiagnosis, which further causes mistreatment with iatrogenic injury and waste of resources. Therefore, we recommended clinicians to be more vigilant about pHLH, and CNS‐HLH should be suspected in those with CNS manifestations and unknown etiology.

## AUTHOR CONTRIBUTIONS

The original manuscript was written by Qi Kong. All authors participated in editing the manuscript, read, and approved the final manuscript.

## CONFLICT OF INTEREST

The authors declare that they have no competing interests.

## ETHICS STATEMENT

This article does not contain any experiments with animals performed by any of the authors. Informed consent was obtained from the patient we reported in the case. All authors agree with publication of this case report and accompanying images
